# Multi-Omics Analysis Reveals Intratumor Microbes as Immunomodulators in Colorectal Cancer

**DOI:** 10.1128/spectrum.05038-22

**Published:** 2023-02-14

**Authors:** Zhi Liu, Xuemei Zhang, Haoding Zhang, Hong Zhang, Zhongyuan Yi, Qingqing Zhang, Qisha Liu, Xingyin Liu

**Affiliations:** a Department of Pathogen Biology—Microbiology Division, State Key Laboratory of Reproductive Medicine, Key Laboratory of Pathogen of Jiangsu Province, Key Laboratory of Human Functional Genomics of Jiangsu Province, Center of Global Health, Nanjing Medical University, Nanjing, China; b The Affiliated Suzhou Hospital of Nanjing Medical University, Suzhou Municipal Hospital, Gusu School, Nanjing Medical University, Nanjing, China; University of Florida

**Keywords:** colorectal cancer, immune-cell infiltration, intratumor microbes, survival, treatment response

## Abstract

Recent studies indicated that intratumor microbes are an essential part of the tumor microenvironment. Here, we performed an integrated analysis of genetic, epigenetic, and intratumor microbial factors to unravel the potential remodeling mechanisms of immune-cell infiltration (ICI) and tumorigenesis of colorectal cancer (CRC). We identified the components and structure of the intratumor microbiome as primary contributors to the difference in survival between ICI subtypes. Multiple tumor-infiltrating immune cells (TIICs) and immune-related genes were associated with intratumor microbial abundance. Additionally, we found that *Clostridium* was enriched in CRC patients who were nonsensitive to immune checkpoint blockade (ICB) therapy. We further provided clues that the intratumor microbes might influence the response to ICB therapy by mediating TIICs, especially MAIT (mucosa-associated invariant T) cells. Finally, three ICB-related TIICs and 22 of their associated microbes showed the potential to predict the response to ICB therapy (area under the receiver operating characteristic curve [AUC] = 89%). Our findings highlight the crucial role of intratumor microbes in affecting immune-cell infiltration patterns, prognosis, and therapy response of CRC and provide insights for improving current immunotherapeutic treatment strategies and prognosis for CRC patients.

**IMPORTANCE** Using the multi-omics data from The Cancer Genome Atlas (TCGA) colorectal cancer (CRC) cohort, we estimated the tumor microenvironment (TME) infiltration patterns of patients and unraveled the interplay of gene expression, epigenetic modification, and the intratumor microbiome. This study suggests the impact of intratumor microbes on maintaining the tumor immune microenvironment in the pathogenesis of CRC and modulating the response to immune checkpoint blockade (ICB) therapy. We identified a set of combined features, including 3 ICB-related tumor-infiltrating immune cells (TIICs) and 22 of their associated microbes, that are predictive of ICB responses.

## INTRODUCTION

The tumor microenvironment (TME) refers to the environment around a tumor, which comprises the extracellular matrix, blood vessels, and cellular players such as immune cells and neurons (1). The tumor-infiltrating immune cells (TIICs) in the TME are primary immune signatures that play critical roles in tumor growth and progression, patient prognosis, and response to immunotherapy (2–4). The immunological component of the TME is a two-edged sword that can either suppress or promote tumor development (5). For example, dendritic cells (DCs) have been reported to be tumor promoting in endometrial carcinoma but tumor suppressive in breast cancer (6, 7). The CD8^+^ tumor-infiltrating lymphocytes repress tumors in a cytotoxic manner and are reported to affect prognosis, immunotherapy response, and survival outcomes in colorectal cancer (CRC) patients (8). Several studies have shown that tumor-associated macrophages (TAMs), a major component of the immune cells in the TME, are associated with response to tumor immunotherapy in CRC (9, 10). A recent study suggested that the tumor immune contexture is a determinant of anti-CD19 CAR (chimeric antigen receptor) T cell efficacy in large B cell lymphoma (11).

CRC is one of the leading causes of cancer-related mortality (12). In addition to the genetic alterations, the development of CRC depends on a close interaction of mutagenized cells with their tumor microenvironment (13). The presence of tumor-infiltrating lymphocytes is associated with a better prognosis in advanced CRC (14). It has been shown that T helper 1 (Th1) cell-mediated immune responses and gamma interferon (IFN-γ) levels in CRC tumors are associated with a better prognosis, whereas Th17 cell-mediated immune responses correlate with a less favorable prognosis, thus highlighting the key role of the T cell response in restricting or driving tumor cell growth (15). Based on this knowledge, the concept of an “immunocore” was established for CRC to make tumor classifications and predict prognosis (16). Approaches targeting various immune-cell types in the TME are currently undergoing trials (17).

TME is affected by various factors, including genetic and epigenetic alterations. For example, KRAS mutations, the most frequent oncogenic driver mutations in human lung cancer cells, impact the lung TME phenotype by non-cell-autonomous modulation of immune cells (18). It was recently reported that extrinsic factors, such as commensal and pathogenic microbiome composition, can, directly and indirectly, alter the behavior of tumor cells and cells within the TME, which also affects disease progression and response to treatment in various tumors (19–21), including CRC (22). For instance, Xing et al. reported that Odoribacter splanchnicus could induce intestinal Th17 cell development and confer resistance against colitis and CRC in wild-type mice (21). Parvimonas micra, a newly identified pathogenic bacterium in CRC, significantly introduced the differentiation of CD4^+^ T cells to Th17 cells and enhanced the oncogenic Wnt signaling pathway in Apc^min/+^ mice (23). An increased abundance of tumor-infiltrating M2-like macrophages was observed in Fusobacterium nucleatum-positive CRC tissues, which conferred on CRC cells a more malignant phenotype (24). Improving our knowledge of how these factors function together to induce tumor-initiating inflammation or to promote tumor growth will help to develop strategies targeting specific inflammatory pathways in the context of CRC.

In this study, using the multi-omics data from The Cancer Genome Atlas (TCGA) CRC cohort, we estimated the TME infiltration patterns of patients and correlated the immune status with genetic, epigenetic, and intratumor microbiome characteristics to unravel their impact on the establishment and maintenance of the tumor immune microenvironment as well as the patients’ clinical prognosis and response to immunotherapy. The results provide clues to elucidate the immune environment of CRC to improve current treatment strategies and prognosis for CRC patients.

## RESULTS

### Landscape of immune-cell infiltration in the TME of CRC.

**FIG 1:**
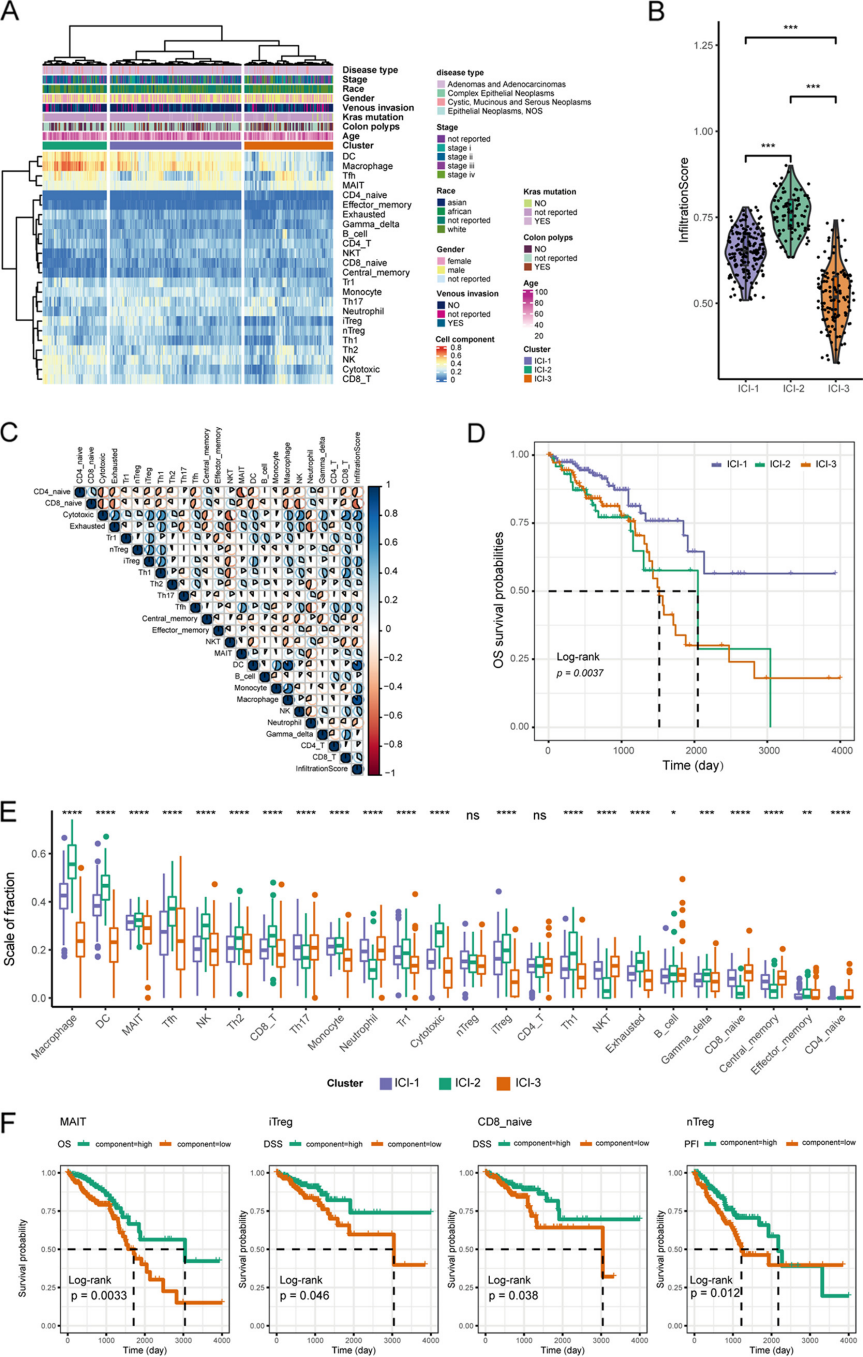
Landscape of immune-cell infiltration in the TME of CRC. (A) Unsupervised clustering of tumor-infiltrating immune cells in TCGA CRC cohorts. (B) Difference in infiltration score among three ICI subtypes. (C) Correlation of the tumor-infiltrating immune cells. Pearson’s correlation analysis between immune cells was performed. The result was visualized using the R package corrplot. The size of the sector area and the gradient of colors represent the correlation coefficient. (D) Kaplan-Meier curves for overall survival (OS) of CRC patients within three ICI subtypes. (E) Fraction of tumor-infiltrating immune cells in three immune subtypes. The statistical difference between three ICI clusters was compared with the Kruskal-Wallis test. *, *P *< 0.05; **, *P *< 0.01; ***, *P *< 0.001. (F) Kaplan-Meier curves of immune-cell types are significantly associated with survival rate.

To evaluate the distinct subtypes of immune-cell infiltration (ICI) in CRC, the TME of CRC was established based on the expression profiles from the TCGA CRC cohort using the ImmuCellAI method (25). Three different immune-cell infiltration patterns were identified with unsupervised clustering, termed clusters ICI-1, ICI-2, and ICI-3 (Fig. 1A; also, see Table S1 in the supplemental material). A significant difference in infiltration score was observed among the three independent ICI subtypes (Fig. 1B). The correlation heat map was generated to visualize the landscape of immune-cell interaction in TME (Fig. 1C).

Impressively, the three independent ICI subtypes had significant survival differences (log rank *P* = 0.0037) (Fig. 1D). To further investigate the intrinsic biological differences that led to distinct clinical phenotypes, we compared the immune-cell compositions of the TME. Subtype ICI-2, with the medium survival rate, was associated with an extremely high level of most subgroups of immune cells, including cells associated with both good and bad prognosis, such as macrophages, DCs, natural killer (NK) cells, CD8^+^ T cells, T helper 2 (Th2) cells, and inducible regulatory T cells (Treg). Subtype ICI-3 could be considered decreased-immunogenicity tumors with a low infiltration level of all immune cells. Compared with ICI-2 and ICI-3, samples in the ICI-1 group showed the best survival rate and exhibited moderate infiltration (Fig. 1E). To further estimate the prognostic value of TIICs, survival analysis was performed based on distinct immune-cell subpopulations. Notably, the infiltration of MAIT (mucosa-associated invariant T) cells was significantly correlated with a longer overall survival time (log rank *P* = 0.003) (Fig. 1F). Thus, the TIIC composition is associated with CRC patients’ survival.

### Identification of immune-gene subgroups.

**FIG 2:**
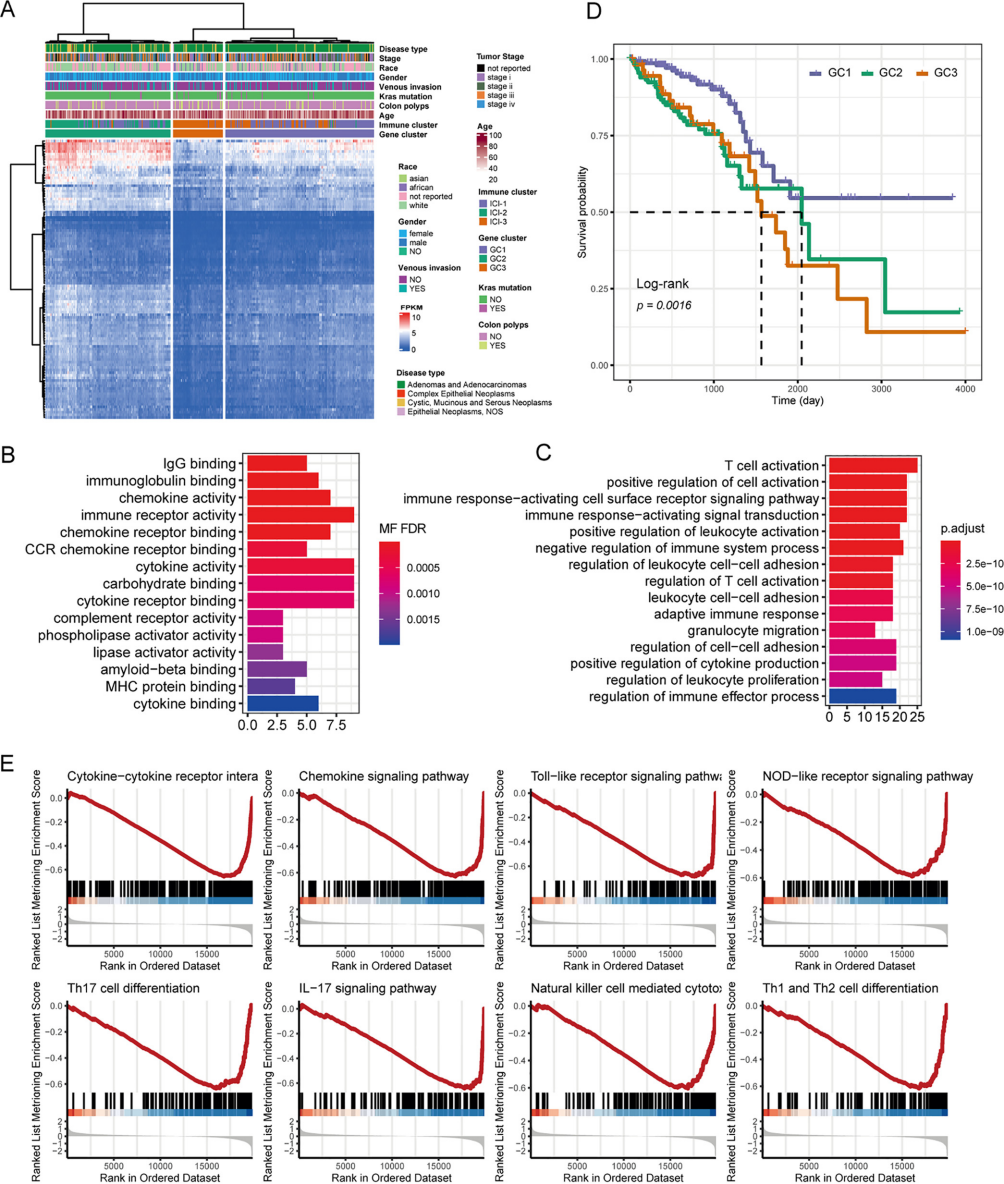
Immunogenic gene subtypes. (A) Unsupervised clustering of DEGs among three ICI subtypes to classify patients into three groups: gene clusters 1 to 3. (B and C) Significantly enriched molecular functions (B) and biological processes (C) of the DEGs were determined by GO analysis. (D) Kaplan-Meier curves for overall survival of CRC patients with three gene subtypes. (E) GSEA analysis revealed the enrichment of immune-related functions for GC1 compared with GC3.

To explore the underlying biological characteristics of distinct ICI patterns, we performed differential analyses to determine the transcriptome variations among these ICI subgroups. We identified 3,561 differentially expressed genes (DEGs) distinguished among the three ICI subtypes. The Boruta algorithm was carried out for the dimension reduction of the ICI gene signatures, resulting in 106 genes that were considered the crucial distinguishing index of different ICI phenotypes. A heat map was created to display the transcriptional distinction among these gene subtypes (Fig. 2A). The ICI clusters were closely correlated with the gene clusters. The phenotype-related genes were significantly enriched in immune-related functions, such as IgG binding, T cell activation, and chemokine activity (Fig. 2B and C).

The three gene clusters were significantly different in survival rates (log rank *P *= 0.0016) (Fig. 2D). Similar to the ICI clustering, the median survival time of gene cluster 1 (GC1) has the best median survival rate. In contrast, gene cluster 3 (GC3) exhibited the worst prognosis. To elucidate the functional difference in gene signature between GC1 and GC3, we explored the biological enrichment by gene set enrichment analysis (GSEA) (Table S2) and found that immune-related functions were enriched in the GC1 subtype (Fig. 2E). The consistency between ICI patterns, transcriptome clusters, and prognosis profiles indicated that the classification of ICI subgroups is reasonable.

### Differences in epigenetic and genetic factors related to the immune phenotype.

**FIG 3:**
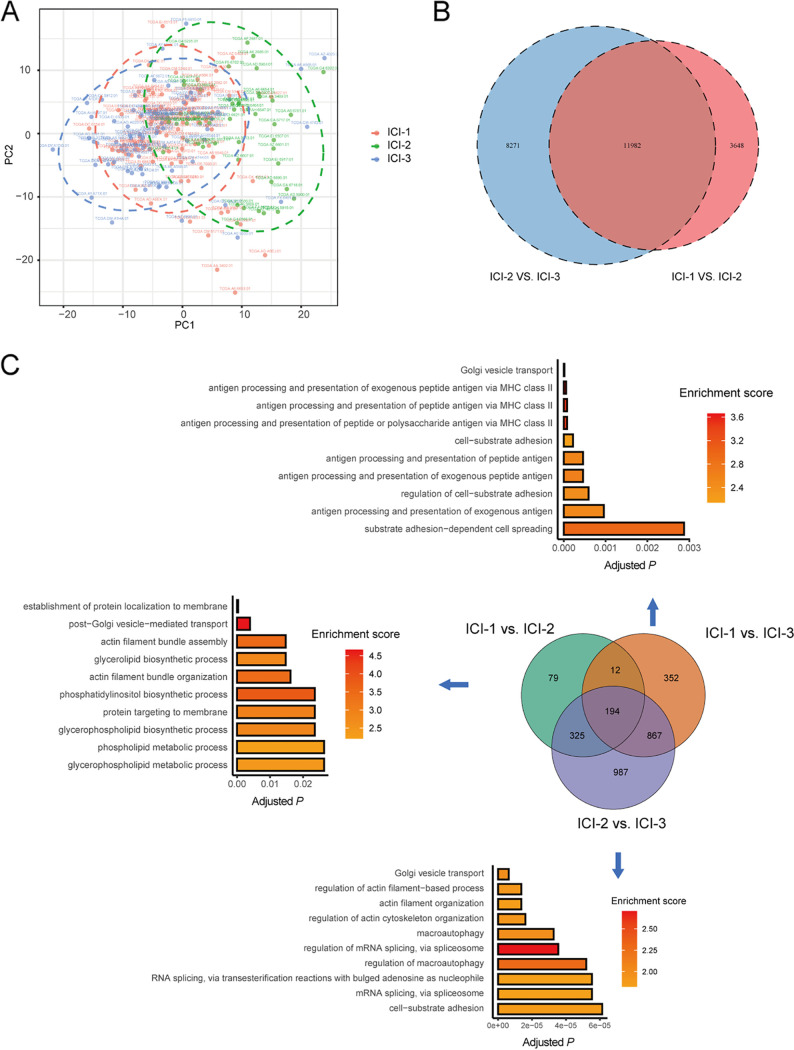
Comparison of epigenetic profiles for three ICI subtypes. (A) PCA plot of DNA methylation profiles of samples in different ICI groups. (B) Overlap of the pairwise differentially methylated CpG probes. No overlaps were observed between the ICI-1 and ICI-3 subtypes. (C) Overlap and functional enrichment of pairwise DEAS events among ICI subtypes.

To gain insights into the underlying mechanisms of distinct immune phenotypes, we further investigated the epigenetic and genetic differences among different ICI subtypes. DNA methylation plays a crucial role in regulating antitumor immunity, the response to immunotherapy, and prognosis (26). We then explored DNA methylation’s role in regulating immune subgroups. First, we analyzed global methylation data available for the TCGA CRC cohort. Principal-component analysis (PCA) revealed no significant DNA methylation difference between ICI-1 and ICI-3 (Fig. 3A). We then determined the groupwise differential CpG sites with the R package ChAMP. In total, 15,630 and 20,253 differentially methylated probes were defined in comparing ICI-1 and ICI-2 subtypes and ICI-2 and ICI-3 subtypes. However, no differentially methylated sites were observed between ICI-1 and ICI-3 subtypes (Fig. 3B). These results suggested that DNA methylation was not a contributor to the difference in prognostic potential between immunity subtypes ICI-1 and ICI-3.

Alternative splicing (AS) is one of the most critical mechanisms of posttranscriptional regulation. AS plays a vital role in immune microenvironment formation (27), which may affect immune-cell infiltration and regulate tumor-associated immune cytolytic activity. Moreover, cancer-specific AS changes have also been recognized as essential signatures to predict treatment efficacy. Therefore, we further identified pairwise differential alternative splicing (DEAS) between ICI subgroups. Functional enrichment analysis showed that the differentially spliced genes between the ICI-1 and ICI-3 subtypes were significantly enriched in the biological process related to antigen processing and presentation (Fig. 3C). These results indicated that the ICI subtype-specific AS of immune-related genes might be involved in the immune-cell infiltration.

**FIG 4:**
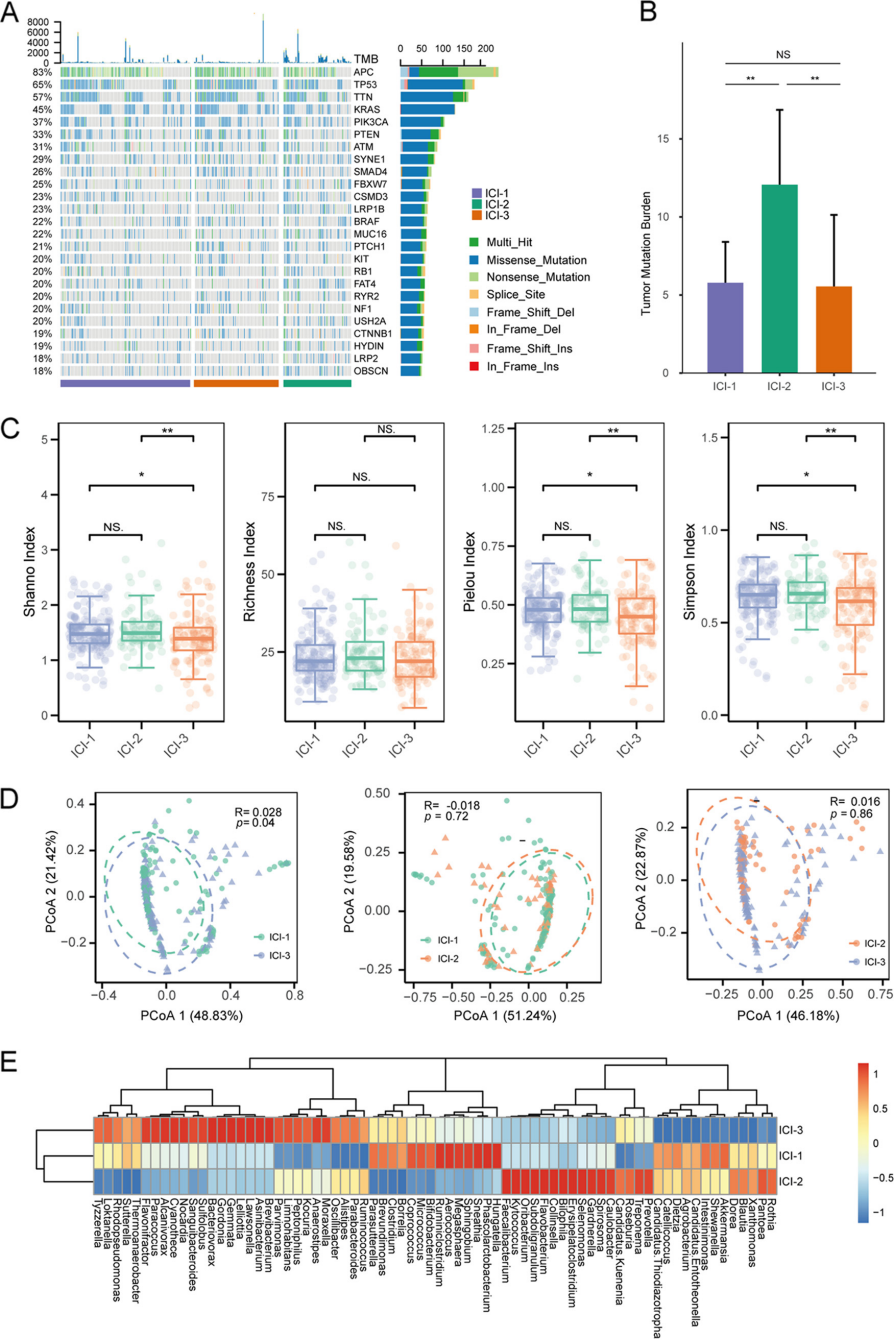
Differences in somatic mutation and intratumor microbiome profiles between ICI subtypes. (A) oncoPrint of each sample in separate ICI subtypes. (B) Differences in TMB between ICI groups. NS, not significant; **, *P *< 0.01 (Wilcoxon test). (C) Comparison of alpha diversity in three ICI subtypes. NS, not significant; *, *P *< 0.05; **, *P *< 0.01 (Wilcoxon test). (D) Beta diversity analysis revealed a significant difference between ICI-1 and ICI-3. (E) Heat map of the differential microbial genera in the pairwise comparison among ICI subtypes. *P* < 0.05 (Wilcoxon test).

Higher tumor mutation burden (TMB) is related to higher numbers of potentially immunogenic neoantigens that may facilitate antitumor immune responses. A myriad of evidence has demonstrated that a high TMB is correlated with the activation of infiltrating CD8^+^ T cells, which recognized tumor neoantigens and killed tumor cells (28). Thus, we speculated that TMB might differ among the ICI subtypes. The somatic mutations in each sample were assessed, and the top 25 genes with the highest alteration frequency are shown in Fig. 4A. The ICI-2 subtype harbored the highest TMB compared with ICI-1 and ICI-3 (*P* < 0.001) (Fig. 4B). Unexpectedly, the TMB in ICI-1 and ICI-3 subtypes, which were most significantly discriminated in survival time, were comparable (Fig. 4B). In addition, the TMB was not correlated with survival time (Fig. S1), which was in line with the previous studies that indicated TMB is not an independent survival biomarker (29). These outcomes suggested novel mechanisms irrelevant to the mutation burden involved in the tumor immune-cell infiltration and difference in prognostic potential between the ICI-1 and ICI-3 subtypes.

### Role of the intratumor microbiome in immunity subtype.

Based on the above analysis, no genomic or epigenomic differences were observed between the most prognosis-discriminate ICI subtypes, i.e., ICI-1 and ICI-3. We then hypothesized that the intratumor microbiome might play a role therein. We obtained the intratumor microbiome profile of the TCGA CRC cohort (Table S3). Diversity analysis revealed that the microbiome was similar between ICI-1 and ICI-2, and both were significantly different from that of ICI-3 (Fig. 4C and D). The alpha diversity, which represents the structure of an ecological community concerning its richness (number of taxonomic groups), evenness (distribution of abundances of the groups), or both, was lowest in the ICI-3 subtype (Fig. 4C). Additionally, beta diversity, which is defined as the dissimilarity among communities, showed a significant difference between ICI-3 and ICI-1 (Fig. 4D).

**FIG 5:**
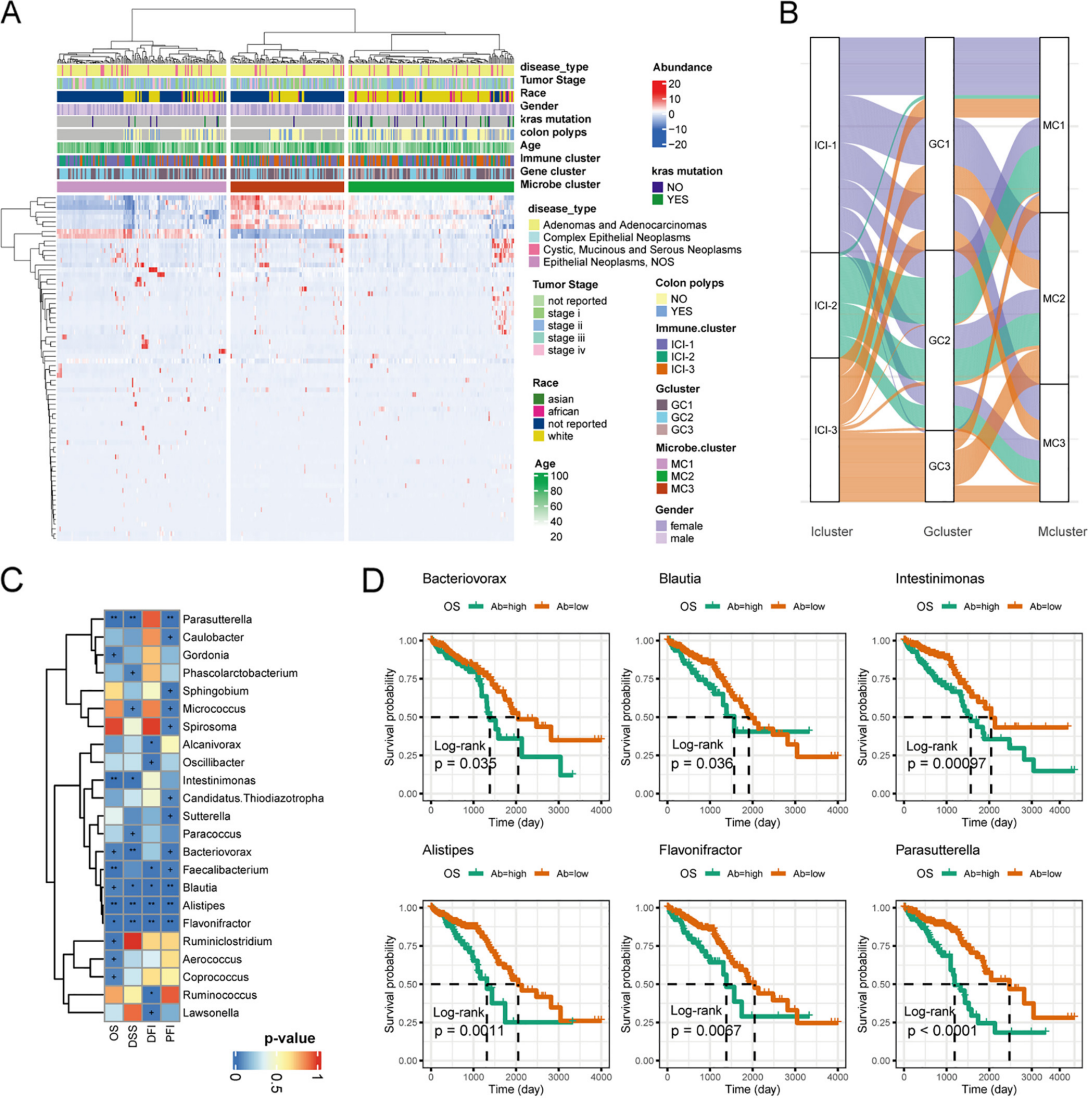
Relationship between the microbiome and immune/gene subtypes and clinical features. (A) Unsupervised clustering of differential microbial taxa among ICI subtypes to classify patients into three microbial groups. (B) Alluvial diagram of ICI subtype distribution in groups with different gene and microbial clusters. (C) Association between microbial abundance and survival rate of patients. +, *P* < 0.05; *, *P* < 0.01; **, *P* < 0.001. (D) Kaplan-Meier curves of example microbes for CRC patients’ overall survival (OS).

We then applied the Mann-Whitney test to determine the microbial signatures among the ICI groups (Fig. 4E). The results showed that multiple known conditional pathogenic bacteria were enriched in ICI-3, including *Parvimonas*, *Alistipes*, *Oscillibacter*, and *Tyzzerella*. In contrast, several commensal beneficial bacteria, such as *Blautia* and *Akkermansia*, were depleted in the ICI-3 subtype. Three microbial clusters were identified using unsupervised clustering (Fig. 5A). The distribution of the patients in the three ICI clusters, gene clusters, and microbial clusters was represented by a Sankey plot (Fig. 5B).

Kaplan-Meier survival analyses based on the microbial abundance were performed to estimate the prognostic value of subtype-specific microbes further. Eleven microbes were associated with survival time (Fig. 5C and D), including *Alistipes* and *Flavonifractor* (Fig. 5D), two taxa associated with CRC. Taken together, these analyses implied a possible role of microbes underlying the difference in prognostic potential between ICI-1 and ICI-3.

### Association between tissue microbes and TIICs.

**FIG 6:**
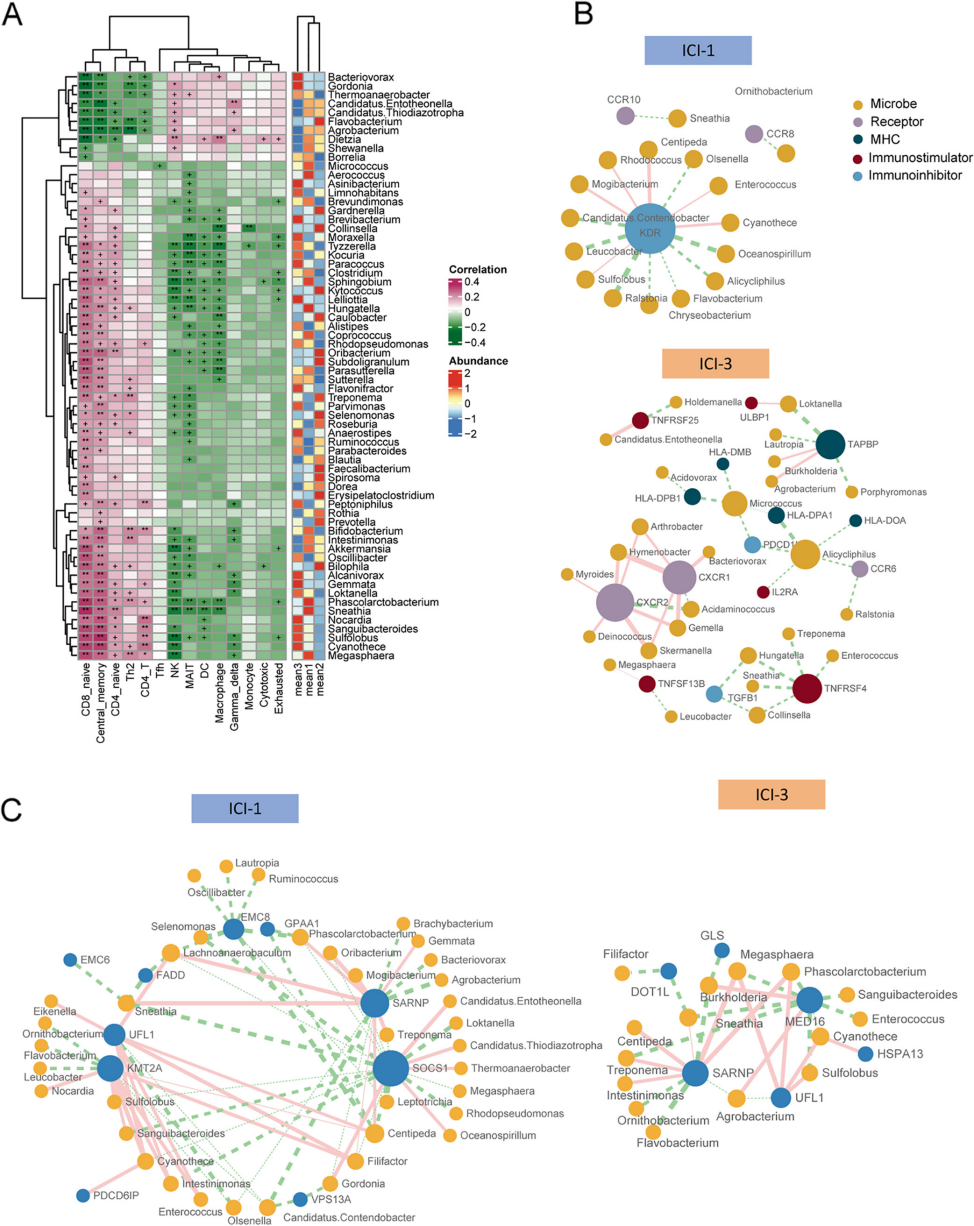
Intratumor microbes interacted with immune features. (A) (Left) Correlation between microbial abundance and immune-cell components. +, *P* < 0.05; *, *P* < 0.01; **, *P* < 0.001 (Spearman correlation). (Right) Average abundance in three ICI subtypes of each microbe. (B) Association between immunomodulator genes and microbes. Associations with Spearman correlation coefficients of >0.3 and FDRs of <0.05 are shown. (C) Association between CTL evasion genes and microbes. Associations with Spearman correlation coefficients of >0.5 and FDRs of <0.05 are shown. Red and green lines indicate positive and negative interactions, respectively.

The above-described analyses indicated a significant role of intratumor microbes in the immune subtype and prognosis. Thus, we speculate that the intratumor microbes might exert effects on the tumor immune microenvironment. Spearman correlation was performed between the microbial abundance and the immune-cell component. Fourteen immune-cell types were significantly correlated with multiple microbes (Fig. 6A). For example, *Alistipes*, enriched in the ICI-3 subtype and predicted poor survival, was negatively correlated with several antitumor immune cells, including NK cells, macrophages, and MAIT cells, and positively associated with CD8 naive cells and memory cells. The pathogenic bacterium recently identified in CRC, *Parvimonas* (30, 31), was negatively associated with NK and MAIT cells and positively associated with naive CD4 and CD8 cells, central memory cells, and Th2 cells. Another significant indicator of CRC, *Bilophila* (32), was negatively correlated with NK cells, MAIT cells, macrophages, and cytotoxic T cells. *Bilophila* species are hydrogen sulfide (H_2_S) producers. Excessive production of H_2_S by the human microbiome has been linked with decreased mucosal integrity and genotoxicity (33) and correlated with increased CRC risk (32, 34).

To confirm the association between tissue microbes and immune-cell components, we applied the association analysis on the coupled single-cell RNA sequencing and 16S rRNA sequencing data for human colon tissues provided by James et al. (35). Seventy significant associations between 19 genus and 20 immune-cell subtypes were observed (Fig. S2; Table S4). The negative association between *Bilophila* abundance and NK cell proportion was reproduced. As should be noted for further classification of immune cells into subpopulations, we observed an opposite association between specific microbes and immune-cell subpopulations. For example, the abundance of *Alistipes* was not related to the portion of total Treg cells in bulk sequencing data of the TCGA CRC cohort; however, it was positively associated with the lymphoid tissue-like Treg (LT-Treg) cell population but negatively associated with the cycling Treg cell population in the single-cell RNA sequencing data.

### Microbes might affect TIIC by regulating the expression and alternative splicing of immune-related genes.

We next determined how the intratumor microbes affect TIICs. We tested the association between microbes and immune-related genes. Correlation analysis was first performed between microbes and 152 immune-modulator genes to explore the modulation of microbes on host immune response. There were 27 genes associated with at least one microbe (Fig. 6B). In ICI-1, microbes were most extensively associated with the kinase insert domain receptor (KDR), an immune inhibitor. In comparison, in ICI-3, associations were observed between microbes and all categories of immune modulators, which indicates a more extensive interaction between microbes and the immune modulators in ICI-3 than ICI-1.

Cancer cells can acquire phenotypic changes that allow them to evade recognition and destruction by immune system cells such as cytotoxic T lymphocytes (CTLs). The cancer-intrinsic CTL evasion genes mediate this process, and significant positive correlations were observed within these genes for tumor immune-cell infiltration (36). We next explored the correlation between microbial abundance with 182 core cancer-intrinsic CTL evasion genes in ICI-1 and ICI-3. Our network analysis identified significant microbe-host gene interactions in the ICI-1 group (Fig. 6C). For example, SOCS1, a major intracellular negative checkpoint of adoptive T cell response (37), exhibited one of the most extensive relationships with microbes. SOCS1 formed the strongest negative association with multiple ICI-1 subtype-enriched genera, i.e., *Phascolarctobacterium*, *Sneathia*, and *Intestinimonas*. In contrast, the interaction network was much simpler in the ICI-3 group (Fig. 6C).

The microbiome also has a role in affecting the alternative splicing of host genes. For example, products from Pseudomonas spp. and *Streptomyces* spp. have been reported to inhibit host splicing machinery (38); the alternative splicing events upregulated in patients with inflammatory bowel disease (IBD) were mapped to the KEGG pathways for “bacterial invasion of epithelial cells” and “pathogenic E. coli infection” (39). Thus, we further investigated whether the ICI subtype-associated microbes affected the alternative splicing of immune-related genes. The DEAS events between ICI-1 and ICI-3 and ICI-1 and ICI-2 subtypes were comprehensively related to microbes (Fig. S3A). However, the DEAS events between ICI-1 and ICI-2 were not (Fig. S3A).

As we demonstrated above, the DEAS events between ICI-1 and ICI-3 subtypes were significantly enriched in the biological process related to antigen processing and presentation. We then examined the correlation between the alternative splicing of genes that fall into this category and microbes (Fig. S3B). Twenty-nine splicing events were significantly associated with 23 microbes that differ between ICI-1 and ICI-3 subgroups. For example, the ICI-1 subtype deletion genus *Oscillibacter* was most negatively correlated with one exon skip (ES) event of CD74, which plays a critical role in processing major histocompatibility complex (MHC) class II molecules in antigen-presenting immune cells. These results indicated that the ICI subtype-specific microbes might influence antigen processing by regulating the alternative splicing of immune-related genes, thus impacting antitumor immunity.

### Difference in ICB response among ICI subtypes.

**FIG 7:**
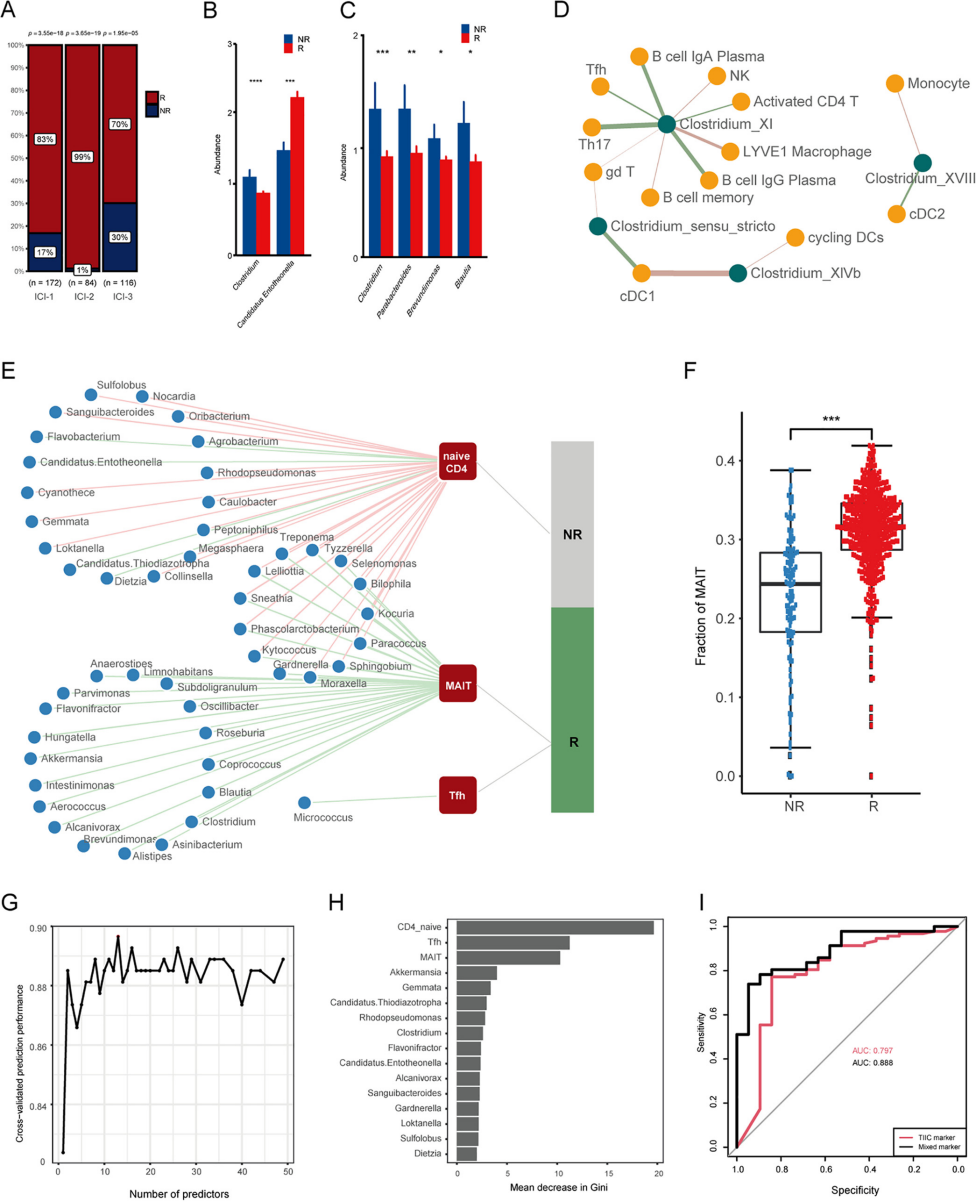
Immune cell infiltration patterns characterized according to ICB response. (A) Percentage of ICB response samples in three ICI subtypes. The statistical difference between ICI clusters was compared with the Kruskal-Wallis test. (B) Differential microbes between responders (R) and nonresponders (NR). (C) Differential microbes between responders (R) and nonresponders (NR) in ICI-1. (D) Association between *Clostridium* and immune-cell components in the scRNA sequencing data set. (E) Correlation between intratumor microbes, TIICs, and ICB response. (F) Proportion of MAIT cells in R and NR groups. (G) Classification performance of a random forest model assessed by the random forest package in R. The cross-validated prediction performance of models with a sequentially reduced number of predictors was explored and ordered by importance. (H) The 25 most discriminant features in the models classify responsive and nonresponsive patients. Bar length indicates the importance of each variable. (I) ROC curves for classifiers based on TIIC markers and mixed markers. AUC, area under the curve.

The composition of the TME has been shown to influence patients’ response to immune checkpoint blockade (ICB). Next, we investigated the correlation between the ICI clusters and immunotherapy response. The ICB therapy response was estimated based on the immune-cell abundance by ImmuCellAI. As shown in Fig. 7A, most of the samples in ICI-2 were sensitive to ICB therapy. Additionally, the ICI-1 subtype exhibits a significantly higher portion of sensitive samples than the ICI-3 subtype (Fig. 7A).

Furthermore, we compared the expression of six essential immune checkpoint blockade-related genes (CTLA4, DIO1, PD1, TIM3, PD-L1, and PD-L2) among the three ICI subtypes. Similarly, we observed that the ICI-2 subtype was marked by a significantly highest expression of all the ICB-related genes. In contrast, the ICI-3 subtype exhibited a lower expression level than ICI-1 and ICI-2 for all ICB-related genes except for DIO1 (Fig. S4). These results suggest that the ICI-3 subtype might be the least suitable for immunotherapy.

### Effects of intratumor microbes on the response to ICB therapy.

Based on the above analysis that microbes involved in the ICI subtype through modulating immune-related genes, we further explored the role of intratumor microbes in patients’ response to ICB treatments. The microbial difference between the responder (R) and nonresponder (NR) was identified. *Clostridium* and a “*Candidatus* Entotheonella” species were enriched in the NR and R groups, respectively (Fig. 7B). Previous studies have suggested that multiple *Clostridium* species have been reported to be more abundant in nonresponders among renal cell carcinoma patients undergoing anti-PD-1 monoclonal antibody (MAb) treatment (40).

Considering the possible differential interactions between genetic, epigenetic, and other environmental factors entangled in the association between microbes and the ICB response, we then conducted clusterwise analysis. A microbial difference between the NR and R groups was observed only in the ICI-1 subtype. The abundance of four microbes was significantly higher in the nonresponders (Fig. 7C), including *Parabacteroides*, *Clostridium*, *Brevundimonas*, and *Blautia*. Our TCGA and small cytoplasmic RNA (scRNA) data analysis also revealed an intensive association between *Clostridium* species and immune-cell subpopulations (Fig. 6A and 7D).

In order to explore the possible mechanisms of intratumor microbes affecting the therapeutic effect of ICB, we analyzed the association between intratumor microbes and ICB-related genes. However, few associations were observed (Table S5). We then tested the association between TIICs and ICB therapy response. The proportion of three immune infiltrating cells, i.e., MAIT cells, naive CD4 cells, and Tfh cells, was significantly different between the NR and R groups (Fig. 7E). As we demonstrated previously, MAIT cells, which are microbe-associated TIICs, were most positively associated with prognosis. We observed a significantly higher portion of MAIT in the responders (Fig. 7F). Accordingly, three of four NR-enriched microbes were negatively associated with the proportion of MAIT cells (Fig. 7E). In conclusion, our findings suggest that aside from affecting the ICB-related genes directly, the intratumor microbes might influence the response to ICB therapy via the mediation of TIICs, especially MAIT cells.

### Identification of markers for ICB response.

The associations between the TIIC portion and ICB responses led to the hypothesis that the proportion of TIICs might indicate therapy outcomes. To explore this notion, random forest classifier models were constructed based on MAIT, naive CD4, and Tfh cells to predict the ICB responses of 372 patients. The prediction model showed a discriminatory power to predict ICB response of 0.797 (area under the receiver operating characteristic [ROC] curve [AUC] of 0.797).

To further refine the signature, we assessed the power of mixed markers of TIICs and microbes to predict the patients’ response to ICB therapy by assessing the differential TIICs and their associated microbes. Feature selection using random forest (RF) machine learning revealed that 25 features, including three TIICs and 22 associated microbes, achieved an increased predictive power (AUC of 0.888) (Fig. 7G to I).

The microbial features with the greatest predictive power for identifying responsive patients included well-known pathogens and probiotics, such as *Akkermansia*, *Clostridium*, and *Flavonifractor*. The bacterium with the highest importance was *Akkermansia*. *Akkermansia* is reported to enhance antitumor immune-cell infiltrates in patients and is associated with improved ICB responses in non-small cell lung cancer and epithelial tumors (41–43). Collectively, our results indicate the potential power of intratumor microbes and TIICs in predicting responses to ICB in CRC patients.

## DISCUSSION

Studies have emphasized that the tumor-infiltrating immune cells in the TME can modulate tumor progression and affect clinical prognosis and are attractive therapeutic targets (44). Remodeling the tumor-immune microenvironment has become a promising strategy to enhance the antitumor immune response in tumors (45). However, the mechanisms underlying the difference in tumor cell infiltration remain unclear. Mutation burden and epigenetic and transcriptional factors have been reported as contributors to regulating the TME (46, 47). Recent evidence has revealed the importance of intratumor microbes for local antitumor immunity (48, 49). In this study, we systematically explored the role of genetic factors, epigenetic factors, and the intratumor microbiome in remodeling the TME and affecting the prognosis of CRC. Our results revealed a crucial role of intratumor microbes in the tumor-infiltrating immune-cell patterns and suggested possible immunotherapeutic strategies for modulating the intratumor microbiome in immunotherapy for CRC.

In the present study, we estimated the TME infiltration patterns in the TCGA CRC cohort with ImmunCellAI. Three different immune-cell infiltration patterns were identified, which represent immunologically “hot,” “moderate,” and “cold” tumors. The samples with moderate immune-cell infiltration were those with the best survival. Then, we correlated the immune status with genetic, epigenetic, or intratumor microbial characteristics to unravel their impact on establishing and maintaining the tumor immune microenvironment in the pathogenesis of CRC. We observed that a high mutation burden was indeed correlated with a high portion of tumor-infiltrating immune cells, which was mostly due to the potential for tumor mutations to generate immunogenic neoantigens. However, in line with the previous reports, the high TMB was not correlated with the patient’s survival (46). On the one hand, this reveals that the strong immune effect introduced by TMB can activate immunity. On the other hand, the mutation itself is harmful and unfavorable to the survival of patients. Consistent with this study, our data indicated that the survival of the “hot” samples was not the best.

We observed a tremendous difference in survival rate between the subtype with “moderate” and “cold” immune infiltration. There was no difference in TMB and DNA methylation. However, they were significantly different in the intratumor microbiome, which indicates that intratumor microbes could affect immune infiltration to a certain extent and that the increase of immune infiltration induced by intratumor microbes might improve the survival of patients. Further association analysis revealed that the intratumor microbes were correlated with various immune-cell components and the expression of many immune-related genes, including immune modulator and tumor escape genes.

Increasing evidence indicates that the microbiota plays a vital role in reshaping immune responses to cancer immunotherapy (50). McCulloch et al. reported two microbial signatures that are associated with favorable and unfavorable clinical responses in melanoma patients treated with anti-PD-1 (51). Sivan et al. revealed that oral administration of *Bifidobacterium* increased dendritic cell activation and improved the tumor-specific CD8^+^ T cell response in the mouse model (52). Moreover, a recent clinical trial showed that live bacterial supplementation led to higher objective response rates and prolonged progression-free survival (PFS) in metastatic renal cell carcinoma patients treated with nivolumab-ipilimumab (53).

In this study, we found multiple ICI-3-associated taxa negatively correlated with patients’ survival, such as *Alistipes* and *Oscillibacter*. *Alistipes* is a relatively new genus isolated primarily from clinical samples and considered a pathogen in colorectal cancer (54). Some studies showed that *Alistipes* might be protective in liver fibrosis, colitis, cancer immunotherapy, and cardiovascular disease. In contrast, other studies indicate that *Alistipes* is pathogenic in colorectal cancer and is associated with mental signs of depression. For example, Moschen et al. (55) showed that Alistipes finegoldii promotes right-sided colorectal cancer via the interleukin 6 (IL-6)/STAT3 pathway. A previous study reported that *Oscillibacter* was increased in mice with ulcerative colitis (UC) and was significantly positively correlated with IL-6 and IL-1β levels (56). In addition, a Mendelian randomization analysis of 3,432 Chinese individuals revealed that increased relative abundances of fecal *Oscillibacter* and *Alistipes* were causally linked to decreased triglyceride concentration (57). In our results, *Alistipes* were negatively correlated with naive CD8^+^ cells and central memory cells and positively correlated with macrophages and MAIT cells. The association between *Alistipes* and macrophages was validated in coupled single-cell sequencing and 16S rRNA sequencing data (Fig. S2). Additional associations between *Alistipes* and immune cells were also observed (Fig. S2), including Th17, CD4^+^ T, and Treg cells. However, *Alistipes* was not related to the gene expression or AS event of any immune-related genes. In contrast, *Oscillibacter* was associated with IgA-producing plasma cells and memory B cells in the single-cell data. In addition, *Oscillibacter* was negatively correlated with a CTL evasion-related gene, i.e., EMC8 (ER membrane protein complex subunit 8) (Fig. 6C), and its abundance was related to the AS event of CD74 (Fig. S3B). These results suggested a regulatory role of *Oscillibacter* on gene expression of immune-related genes, while *Alistipes* might impact immune-cell infiltration indirectly, for example, by affecting the microbial relationships in communities. As we reported previously (58), the association between Alistipes finegoldii and Parvimonas micra, a well-known pathogenic bacterium in CRC, was commonly altered in multiple CRC cohorts.

Understanding the mechanism of microbiota in improving responses to immune checkpoint therapy is key to therapeutically harnessing them for targeted adjuvant therapies. We therefore explored the interaction between the intratumor microbes and ICB-related genes. Few associations were observed. However, the proportion of MAIT cells differs significantly between the NR and R groups. MAIT is the most abundant T cell subset recognizing bacterial compounds (59). The microbiota-derived signals affect all stages of MAIT cell biology, including intrathymic development, peripheral expansion, and functions in specific organs (60). We identified MAIT as the most significant immune cell component associated with CRC patients’ survival, and it correlated with multiple microbes. Growing evidence indicates that MAIT cells influence antitumor immunity against colorectal cancer, though its role in cancer progression is under debate (61). Microbes play crucial roles in regulating the growth of MAIT cells (61). For example, germfree (GF) mice exhibit fewer MAIT cells than animals housed under specific-pathogen-free (SPF) conditions. Hence, our results suggested that the intratumor microbes might influence the response to ICB therapy via the mediation of TIICs, especially MAIT cells. It is necessary to validate our results in additional single-cell sequencing data sets and by experiments to confirm the potential effects of specific microbes on TIICs. An RF model based on the ICB-related TIICs revealed a relatively high discriminatory power to predict the ICB response (AUC = 0.797). Adding 22 TIIC-associated microbes increased the AUC by 9.1% (AUC = 0.888), suggesting a possible prognostic value of the mixed features consisting of TIICs and intratumor microbes.

There are a few limitations to this study. For example, we cannot formally exclude the presence of additional immune-cell subtypes, which modulate the tumor immune microenvironment of CRC but are not assessed by ImmunCellAI. In addition, as revealed by the analysis of scRNA sequencing (scRNA-seq) data, one taxon could be associated with different immune-cell subtypes in opposite directions. Second, contaminants might exist, because the microbial abundance was extracted from RNA-seq or WGS data. It was revealed that species that are equivalent across sample types are predominantly contaminants (62). Though our analysis was based mainly on the differential taxa between compared groups, a few remaining contaminants might still complicate the results. Future studies should expand on this work by including a large cohort with multi-omics data coupled with scRNA-seq and 16S rRNA sequencing or metagenomic sequencing. Based on these data, we can draw a more detailed TIIC landscape and provide a precise intratumor microbiome profile in CRC, and then determine the interplay between microbes and TIICs. To further validate microbes’ role in affecting immune-cell infiltration, *in vitro* and *in vivo* experiments should be conducted.

In conclusion, our study comprehensively explored the ICI landscape of CRC, providing an in-depth interaction scenario of TIIC regulation in CRC, and first reported that the difference in ICI patterns was related to the intratumor microbiome and immunotherapy response. It facilitated the identification of ideal candidates for optimizing immunotherapeutic strategies.

## MATERIALS AND METHODS

### Landscape of immune-cell infiltration in TME.

RNA sequencing data for colon cancer and the corresponding clinical profiles were downloaded from the TCGA database. The gene expression profiles of TCGA CRC in FPKM (fragments per kilobase per million) format were obtained from the TCGA portal. The gene expression profile of the TCGA CRC samples was analyzed by ImmunCellAI (http://bioinfo.life.hust.edu.cn/ImmuCellAI#!/) to obtain a fraction matrix of ICI, which estimated the abundances of 24 distinct leukocyte subsets (25). The response to ICB treatment was also predicted based on the expression profiles by ImmunCellAI.

### Identification of gene features associated with ICI subgroups.

ICI subgroup-related DEGs were determined among the ICI patterns by using the R package DEseq2. The genes with false discovery rates (FDR) of <0.05 and absolute fold changes of >2 were considered significant and retained for further analysis. Gene ontology (GO) annotation and GSEA was conducted with the R package clusterProfiler to elucidate the biological role of DEGs. The Boruta algorithm was applied to perform feature selection on the DEGs with the R package Boruta to refine the gene features further.

### Processing of mutation data.

The somatic mutation information of the TCGA-CRC cohort was obtained from the TCGA database. The R package maftools (63) was used to calculate the number of somatic nonsynonymous point mutations within each sample and plot the oncoplot for each gene for individual samples. The TMB was also calculated with maftools. The difference in TMB between any two ICI subtypes was calculated by Wilcoxon’s rank sum tests.

### DNA methylation analysis.

DNA methylation data from the TCGA colon adenocarcinoma (COAD) and rectum adenocarcinoma (READ) cohorts (Illumina Human Methylation 450k) were downloaded from the TCGA database. Methylation data were normalized, and differentially methylated probes were detected by the R package ChAMP (64). Probes with *q* value of <0.05 were defined as significant.

### Identification of DEAS.

Alternative splicing data from patients with COAD and READ were downloaded from the TCGA SpliceSeq database (65). To determine differences in AS events between ICI subgroups, we identified significant DEAS events by Mann-Whitney test based on the percent spliced in (PSI) values for splice events. Splice events with adjusted *P* values of <0.05 were identified as DEAS events.

The association between DEAS events and microbial abundance was calculated with the R package Hmisc, and associations with an absolute correlation of more than 0.6 and FDR less than 0.05 were illustrated with Cytoscape.

### Collection and process of CRC microbiome data.

The normalized microbial abundance profile was provided by Poore et al. (20), who re-examined treatment-naive whole-genome and transcriptome sequencing from the TCGA samples for microbial reads and quantified the microbial abundances. Three hundred seventy-two samples with coupled RNA sequencing and clinical profiles were employed for subsequent analysis. Differential microbes were identified between each of the two ICI subgroups with Mann-Whitney tests. The microbes with adjusted *P* values of <0.05 were significantly different in abundance. Determination of alpha diversities was conducted with the R package vegan. Beta diversity was measured via Bray-Curtis distances and visualized in principal-coordinate analysis (PCoA) plots with the R packages GUniFrac and ggplot2.

### Random forest model.

We implemented an RF model using the R package caret to develop a model capable of distinguishing ICB-nonresponsive versus -responsive control samples. A custom machine-learning process was conducted using features of TIIC proportion and/or microbial abundance. To achieve optimized numbers of a signature set, we employed feature selection with the R package randomForest. In doing so, the discriminatory 3 TIICs and 22 microbes were selected from the top-performing model. Model training was carried out using 70% of the sample set. The important features that contributed to prediction were identified via a 10-fold cross-validation procedure. The ROC curve was obtained with R package pROC to evaluate the constructed model, and the AUC was used to assess the prediction model's discriminative ability.

### Data availability.

The study was based on the data available in The Cancer Genome Atlas (TCGA) database (http://cancergenome.nih.gov). All data relevant to the study are included in the article or the supplemental material. The code for this study is available from the corresponding author upon request.
