# Supplementary material for: Multi-Omics Analysis Reveals Intratumor Microbes as Immunomodulators in Colorectal Cancer
Source: Microbiol Spectr. 2023 Feb 14;11(2):e05038-22. doi: 10.1128/spectrum.05038-22 (PMC10100960; doi:10.1128/spectrum.05038-22)
Supplement: Supplemental file 1 — Supplemental material. Download spectrum.05038-22-s0001.pdf, PDF file, 4.0 MB [file spectrum.05038-22-s0001.pdf]

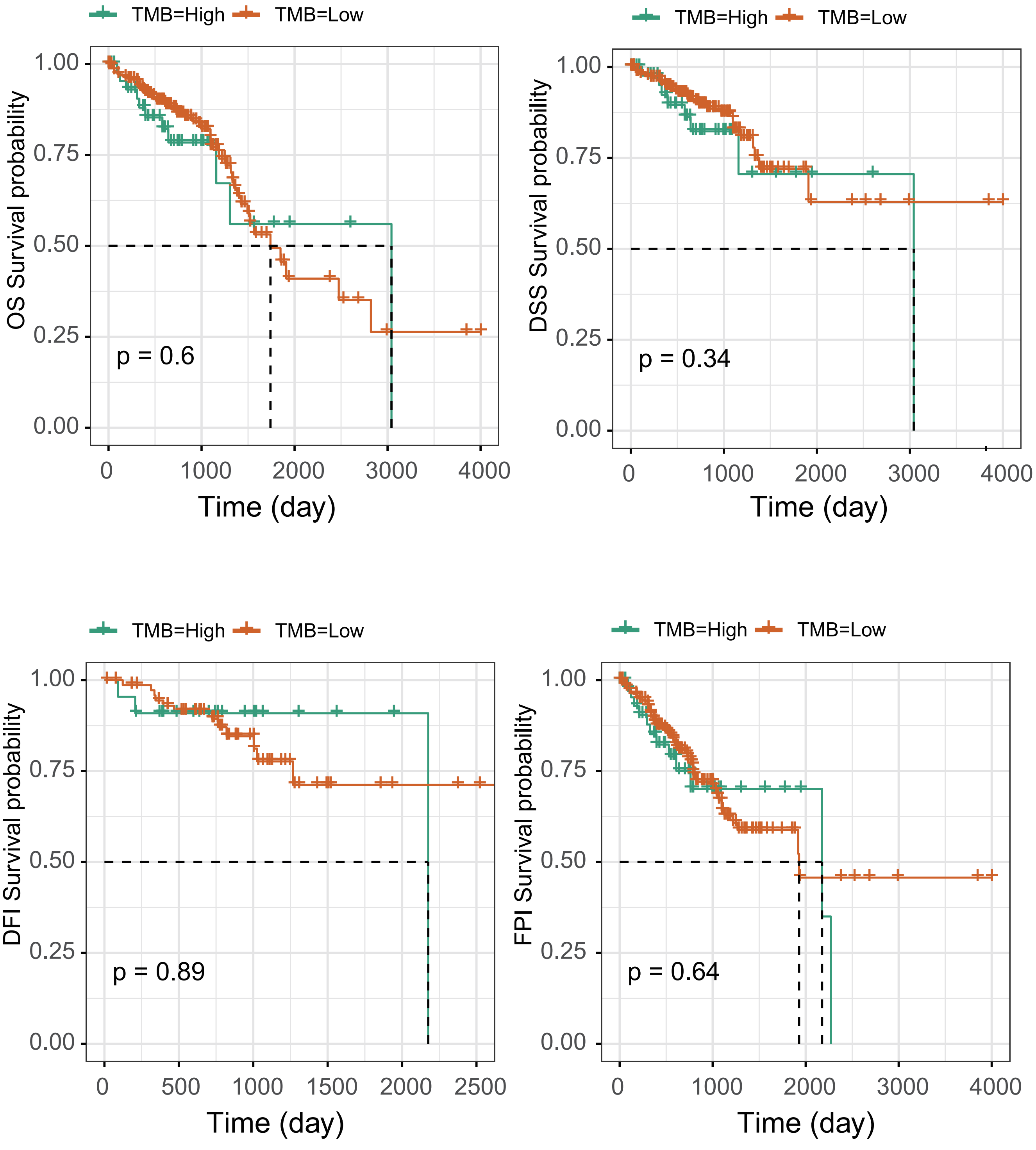
TMB=High
TMB=Low
OS Survival probability
p = 0.6
Time (day)
DSS Survival probability
p = 0.34
DFI Survival probability
p = 0.89
FPI Survival probability
p = 0.64
1.00
0.75
0.50
0.25
0.00
0
500
1000
1500
2000
2500
3000
4000

Figure S1

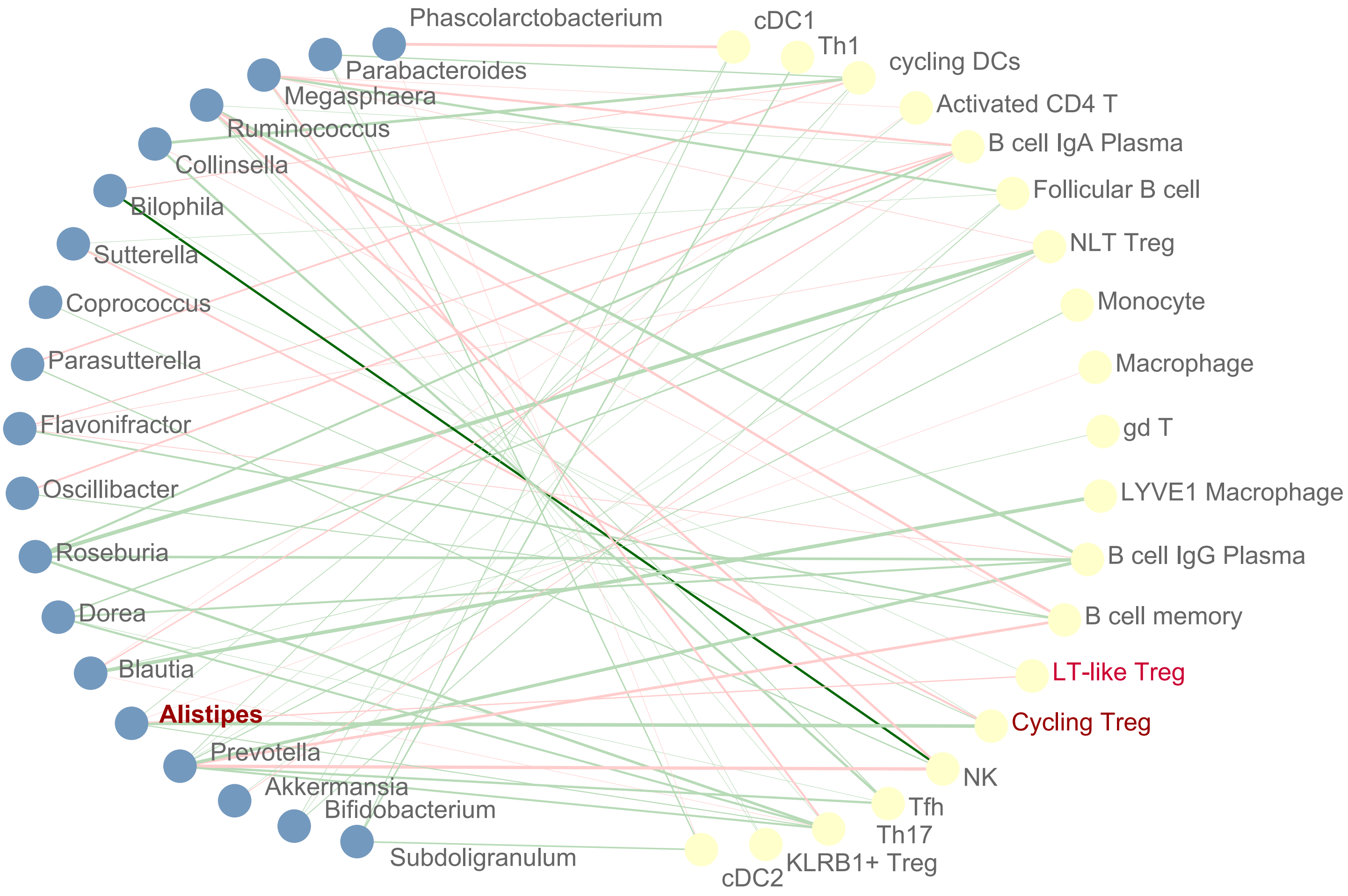
Phascolarctobacterium
Parabacteroides
Megasphaera
Ruminococcus
Collinsella
Bilophila
Sutterella
Coprococcus
Parasutterella
Flavonifractor
Oscillibacter
Roseburia
Dorea
Blautia
Alistipes
Prevotella
Akkermansia
Bifidobacterium
Subdoligranulum
cDC1
Th1
cycling DCs
Activated CD4 T
B cell IgA Plasma
Follicular B cell
NLT Treg
Monocyte
Macrophage
gd T
LYVE1 Macrophage
B cell IgG Plasma
B cell memory
LT-like Treg
Cycling Treg
NK
Tfh
Th17
KLRB1+ Treg
cDC2

Figure S2

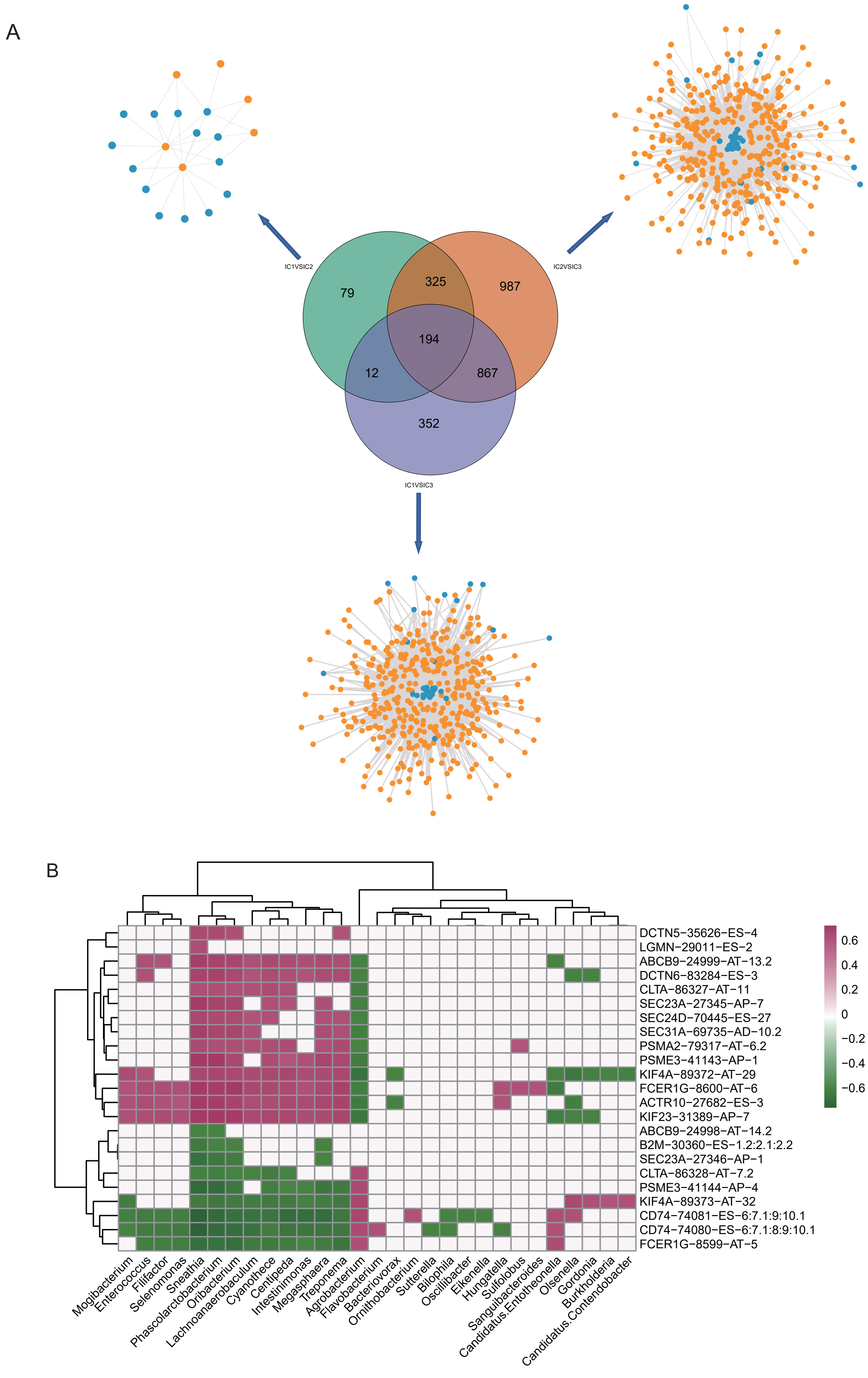
A
IC1VSIC2
IC2VSIC3
IC1VSIC3
79
325
987
194
12
867
352
B
DCTN5−35626−ES−4
LGMN−29011−ES−2
ABCB9−24999−AT−13.2
DCTN6−83284−ES−3
CLTA−86327−AT−11
SEC23A−27345−AP−7
SEC24D−70445−ES−27
SEC31A−69735−AD−10.2
PSMA2−79317−AT−6.2
PSME3−41143−AP−1
KIF4A−89372−AT−29
FCER1G−8600−AT−6
ACTR10−27682−ES−3
KIF23−31389−AP−7
ABCB9−24998−AT−14.2
B2M−30360−ES−1.2:2.1:2.2
SEC23A−27346−AP−1
CLTA−86328−AT−7.2
PSME3−41144−AP−4
KIF4A−89373−AT−32
CD74−74081−ES−6:7.1:9:10.1
CD74−74080−ES−6:7.1:8:9:10.1
FCER1G−8599−AT−5
0.6
0.4
0.2
0
−0.2
−0.4
−0.6
Mogibacterium
Enterococcus
Filifactor
Selenomonas
Sneathia
Phascolarctobacterium
Oribacterium
Lachnoanaerobaculum
Cyanothece
Centipeda
Intestinimonas
Megasphaera
Treponema
Agrobacterium
Flavobacterium
Bacteriovorax
Ornithobacterium
Sutterella
Bilophila
Oscillibacter
Eikenella
Hungatella
Sulfolobus
Sanguibacteroides
Candidatus.Entotheonella
Olsenella
Gordonia
Burkholderia
Candidatus.Contendobacter

Figure S3

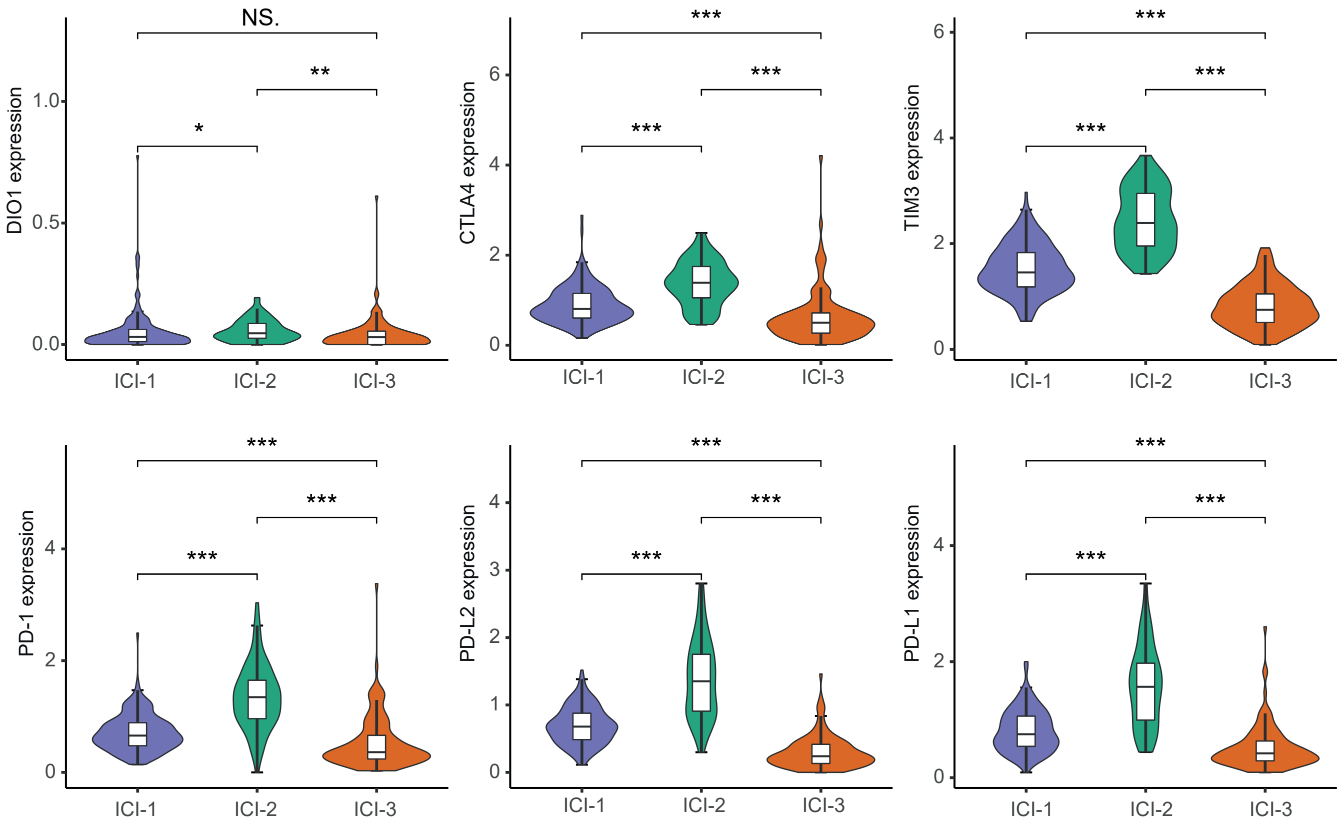

DIO1 expression
NS.
**
*
ICI-1
ICI-2
ICI-3
CTLA4 expression
***
TIM3 expression
PD-1 expression
PD-L2 expression
PD-L1 expression


Figure S4

## Supplementary Figure Legends

**Figure S1. The correlation between tumor mutation burden (TMB) and patients' survival time.** Kaplan-Meier curves for overall survival (OS), disease-specific survival (DSS), disease-free interval (DFI), and progression-free interval (PFI) of CRC patients with low or high TMB.

**Figure S2. Associations between microbes and immune cell components based on the analysis of coupled scRNA sequencing and 16S rRNA sequencing data of human colon tissues.** The red and green edges represent positive and negative correlations, respectively. The width of edges is in proportion to the strength of correlation. Associations with a spearman *p* value less than 0.05 were presented.

**Figure S3. The comparison of differential alternative splicing events (DEAS) among three ICI subtypes.** (A)The association of microbial abundance with DEASs between ICI-1 and ICI-2, ICI-2 and ICI-3, and ICI-1 and ICI-3. (B) The correlation between microbe and DEAS genes related to antigen processing and presentation.

**Figure S4. The difference in six key immune checkpoints blockade (ICB)-related genes in three ICI subtypes.** The comparison of gene expression (FPKM) of ICB-related genes among ICI-1, ICI-2, and ICI-3 (Wilcoxon test, *$p < 0.05$; **$p < 0.01$; ***$p < 0.001$).
